# Factors associated with the interruption of antiretroviral therapy among people living with HIV/AIDS in Brazilian municipalities between 2019 and 2022

**DOI:** 10.1590/1980-549720250015

**Published:** 2025-04-07

**Authors:** Ana Paula da Cunha, Jurema Corrêa da Mota, Marly Marques da Cruz, Raquel Miranda, Liza Rosso, Mariele Kruppa, Juliane Santos, Andreia Souza Pinto da Silva, Giordana Faccin, Caroline Schweitzer, Ronaldo Zonta, Vanda Lúcia Cota

**Affiliations:** I Fundação Oswaldo Cruz, Sergio Arouca National School of Public Health - Rio de Janeiro (RJ), Brazil.; II Fundação Oswaldo Cruz, Institute of Scientific and Technological Communication and Information on Health - Rio de Janeiro (RJ), Brazil.; III Secretaria Municipal da Saúde de Curitiba - Curitiba (PR), Brazil.; IV Secretaria Municipal de Saúde de Campo Grande - Campo Grande (MS), Brazil.; V Secretaria Municipal de Saúde de Florianópolis - Florianópolis (SC), Brazil.

**Keywords:** Acquired immunodeficiency syndrome, Antiretroviral therapy, highly active, Withholding treatment, Treatment adherence and compliance, Barriers to access of health services

## Abstract

**Objective:**

To analyze the factors associated with antiretroviral therapy interruption among people living with the human immunodeficiency virus participating in the *A Hora é Agora* [The Time is Now] project in the municipalities of Campo Grande, Curitiba, and Florianópolis.

**Methods:**

This is a cross-sectional analytical study using data from the Brazilian Medication Logistics Control System, collected between October 2019 and September 2022. Bivariate analyses were performed to assess associations between sex, age group, race/skin color, and level of education, with the outcomes of treatment interruption and the number of times the treatment was interrupted, using the χ^2^ test with a 5% significance level.

**Results:**

In Campo Grande, treatment interruption was more frequent among individuals with eight to 11 years of formal education (34.0%). In Curitiba, women (20.4%) showed a higher frequency of treatment interruption, and the age group of 50 years or older had a higher likelihood of treatment interruption (OR: 1.73; 95%CI: 1.12-2.66). In Florianópolis, 31.1% of women experienced treatment interruption, and individuals with up to seven years of formal education had a higher likelihood of treatment interruption (OR: 1.62; 95%CI: 1.15-2.29).

**Conclusions:**

The interruption of antiretroviral therapy was significantly associated with sex, level of education, and age group, with distinct patterns across the analyzed territorial contexts. These findings highlight the need for targeted interventions aimed at vulnerable groups, considering regional differences and local challenges to improve treatment adherence.

## INTRODUCTION

Despite efforts undertaken on a global scale to combat the human immunodeficiency virus (HIV)/acquired immunodeficiency syndrome (AIDS) through advances in prevention strategies and antiretroviral therapy (ART), the infection continues to pose a substantial public health challenge worldwide^1^.

Brazil has ensured universal and free access to ART since 1996^2^, which is indicated for all people living with HIV (PLHIV), regardless of their immunological status. When properly administered, ART demonstrates efficacy in suppressing virus replication^3^; however, it does not provide a definitive cure, and in case of treatment interruption, the virus regularly regains its activity in weeks^4^.

Since the 1980s, there have been about 20.8 million deaths from HIV/AIDS worldwide and 382,521 in Brazil^2^ ^,^ ^5^. Currently, about 39.8 million people are living with the infection/disease worldwide, and of these, 29.8 million are under treatment^5^. In Brazil, there are approximately 990 thousand PLHIV, of which 83% are under treatment, which indicates that, even with universal access to medication, there are still people who do not take it^5^.

The free distribution of medication alone does not guarantee access to treatment, considering that inequalities in access and adherence to health care, the stigma associated with the infection, risk behaviors, and challenges in the implementation of effective prevention strategies contribute to the occurrence of the disease^6^ ^-^ ^8^.

In Brazil, the HIV Care Continuum (*Cascata do Cuidado Contínuo à PVHIV*) was established, covering timely diagnosis, bonding and retention to the service, initiation of ART, and viral control through treatment adherence^9^. Although the use of the medication seems to be an individual action, adherence involves shared responsibilities between the user, the healthcare team, and the support network. This collaboration is crucial to address sociocultural and economic singularities, aiming to improve the quality of life of PLHIV^10^ ^,^ ^11^.

Nonadherence is the main cause of ART failure, occurring when the patient does not consistently follow the therapy regimen, either by not starting the treatment or by interrupting it. This compromises viral suppression, reduces efficacy, and increases the risk of drug resistance and disease progression^10^ ^,^ ^12^ ^,^ ^13^.

Treatment interruptions of PLHIV may be temporary or complete. These patterns can be influenced by sociodemographic, behavioral factors and those associated with access to healthcare services, leading to increased viral load, disease progression, and compromising HIV control^14^ ^,^ ^15^.

These difficulties are particularly evident in Brazilian cities such as Florianópolis (state of Santa Catarina - SC), Curitiba (state of Paraná - PR), and Campo Grande (state of Mato Grosso do Sul - MS), which present specific epidemiological and social challenges, directly impacting the continuity of treatment and adherence to it^16^ ^-^ ^18^. Florianópolis has the third highest HIV/AIDS detection rate in Brazil^2^, facing a scenario that requires continuous control and prevention actions^17^. In Curitiba, treatment adherence challenges are significant among vulnerable groups, due to factors such as stigma and social barriers^2^ ^,^ ^8^. In Campo Grande, difficulties are also evident, particularly in peripheral areas with restricted access to healthcare services^2^ ^,^ ^18^.

Given the need to face treatment interruption, the *A Hora é Agora* (AHA) [The Time is Now] Project was implemented in five Brazilian municipalities, but this study included Curitiba, Porto Alegre, and Florianópolis, as Porto Alegre (state of Rio Grande do Sul - RS) and Fortaleza (state of Ceará - CE) were implemented later and did not have enough data for the analyzed period. The AHA Project is part of a cooperation agreement between the Sergio Arouca National School of Public Health, from Fundação Oswaldo Cruz (Fiocruz), and the Centers for Disease, Control and Prevention (CDC), funded through the US President’s Emergency Plan for AIDS Relief, in partnership with the Department of HIV/AIDS, Tuberculosis, Viral Hepatitis and other Sexually Transmitted Infections of the Brazilian Ministry of Health, the municipal health departments, and civil society organizations. These institutional partners constitute the project’s steering committee, which aims to expand access to prevention, diagnosis, and immediate treatment for HIV/AIDS focused on the most vulnerable key population. To this end, some innovations in services of the Brazilian Unified Health System (SUS) that allow greater efficiency in the intervention were introduced through the project, such as: fast viral load and CD4, fast creatinine, the linker (peer browser) that supports the user to ensure bonding/adherence, the delivery of the self-test and the medication at home (ART and pre-exposure prophylaxis for HIV [PrEP] delivery), teleconsultation, and telePrEP. This project is implemented within the scope of the SUS, that is, it has the guarantee of universal access, but it operates with two strategic components, which are communication, focused on the most vulnerable population that faces frequent access barriers, and monitoring/evaluation, which allows for the monitoring of all existing strategies and the necessary adjustments throughout the implementation.

The assessment of antiretroviral therapy interruption is paramount to monitor the effectiveness of health policies and identify institutional barriers, such as access problems and stigma, allowing the identification of vulnerable patterns and subgroups in order to improve treatment adherence^19^.

We assumed that the interruption of antiretroviral therapy among PLHIV was influenced by sociodemographic characteristics. Taking this into consideration, in this study we aimed to analyze the factors associated with the interruption of antiretroviral therapy among PLHIV participating in the AHA Project in the municipalities of Campo Grande, Curitiba, and Florianópolis, Brazil.

## METHODS

### Study design and population

This is a cross-sectional and analytical study, with units of analysis in the municipalities of Campo Grande, Curitiba, and Florianópolis. PLHIV aged 15 years or older, regardless of gender at birth, who were taking ART between October 2019 and September 2022, the period of the AHA Project, were included. The data were obtained from the Brazilian Medication Logistics Control System (*Sistema de Controle Logístico de Medicamentos* - Siclom), which allows monitoring the dispensation of antiretrovirals.

After ethical approval, the identified data were anonymized and stored in a secure and restricted manner. The database included sociodemographic information and medication dispensations, with completeness greater than 90%. Variables were created for analysis of treatment interruption, defined by the absence of drug retrieval for more than 28 days after the expected date. Interruptions were categorized as none, one to five times, and six to ten times.

### Outcomes

For analyzing the profile of interruptions, all those who, at some point in the period, did not retrieve the medication 28 days after the expected date of ART dispensing were considered as abandonment, and this criterion was applied to all intervals between dispensations in the period. Based on this information, two outcomes were considered:

- Treatment was interrupted (without ART dispensation) at some point (yes, no);
- Frequency of treatment interruption in the study period (none; one to five times; and six to ten times).

### Covariates

The covariates considered were: sex at birth (women, men), age group (15-29, 30-39, 40-49, and 50 years or older), level of education (unknown, up to seven years, from eight to 11 years, and 12 years or over of formal study), and race/skin color (white; Black - sum of Black and mixed-race individuals; Asian/Indigenous - sum of Asian and Indigenous individuals). The aggregation of Black and mixed-race is due to similar phenotypic and social characteristics, while that of Asian, Indigenous, and not informed is due to the low number of observations.

### Statistical analysis

Descriptive analysis and associations were performed separately for Curitiba, Campo Grande, and Florianópolis, using the χ^2^ test to compare the frequencies of sociodemographic variables in relation to the outcomes (5% significance). This sectoral approach allowed for a specific analysis of each location, avoiding the joint evaluation of all participants.

The absolute and relative frequencies of the covariates were compared according to the outcomes using the χ^2^ test. To analyze the associations, two logistic regression models were adjusted: a binomial model and a multinomial one. The choice of models considered the nature of the outcomes and the complexity of the associations, using the lower risk categories as a reference.

Binomial logistic regression was used for dichotomous outcome, estimating the odds ratio in relation to the reference event. Multinomial logistic regression was applied for outcomes with more than two unique categories, allowing for the odds ratios to be estimated for each category. This approach provides a more comprehensive and robust analysis for complex outcomes.

The initial selection of variables for the regression models was based on bivariate analysis, with a cutoff point of p<0.20, to include covariates potentially associated with the outcomes, even without a 5% significance. Subsequent inclusion and exclusion of variables in the final models considered adjustment and parsimony, prioritizing those that significantly contributed to explain the variation of outcomes, according to the Hosmer-Lemeshow test.

The results of the logistic regressions were presented as adjusted odds ratios (OR), with their respective 95% confidence intervals (95%CI). All analyses were conducted in the R programming language (version 4.3.2), using the ggplot2, dplyr, sjlabelled, nnet, and gtsummary packages (https://www.r-project.org/)^20^ ^-^ ^23^.

### Ethical aspects

The research project was submitted to the Scientific Committee of the Centers for Disease Control, the Research Ethics Committee of the Sergio Arouca National School of Public Health/Fiocruz, the National Commission of Ethics in Research, and the Ethics Committees of the municipal health departments of Curitiba, Florianópolis, and Campo Grande in 2022, and it was approved in all instances, with Certificate of Presentation for Ethical Consideration No. 59072922.0.0000.5240, by protocol No. 5.734.909/2022.

## RESULTS

Between October 2019 and September 2022, 15,879 individuals were under treatment for HIV/AIDS in Curitiba, Campo Grande, and Florianópolis. In Curitiba, 72% of the participants were men and 65% identified as white, with 52% having a low level of education (one to seven years of formal education). The predominant age groups were 30 to 39 years (30%) and 40 to 49 years (28%). In the group that did not abandon the treatment, most were men (76%) and white (68%). Conversely, among those who abandoned it, the proportion of women was higher (35%), as well as that of Blacks (38%), predominating the groups of 15 to 29 years old and 30 to 39 years old.

In Campo Grande, 69% of the participants were men and 60% were white, with 55% having up to seven years of formal education. The most common age groups were 30 to 39 years (27%) and 40 to 49 years (26%). In the group that maintained the treatment, 74% were men and 63% were white. Among those who abandoned it, there were more women (38%) and Blacks (42%), with a prevalence in the age group of 15 to 29 years.

In Florianópolis, 68% of the participants were men and 62% were white, and half had between one and seven years of formal education. The most frequent age groups were 30 to 39 years (29%) and 40 to 49 years (25%). In the group without abandonment, 73% were men and 66% were white. Among those who abandoned it, the proportion of women (42%) and Blacks (40%) was higher, with a prevalence in the groups of 15 to 29 years old and 30 to 39 years old.

In the analysis of the association between treatment interruption and sex, we found that women in Curitiba (p=0.030) and Florianópolis (p=0.015) were more prone to interruption. Low level of education was also associated with a higher probability of interruption in Campo Grande (p=0.029) and Florianópolis (p=0.003). In Curitiba, we observed a higher proportion of interruption in the age groups 30 to 39 years (27.7%) and 50 years or older (34.1%) (p=0.012) (Table 1).

**Table 1:** Distribution of sociodemographic characteristics according to treatment interruption in each municipality. Campo Grande (MS), Curitiba (PR), and Florianópolis (SC), from October 2019 to September 2022.

Variables	Treatment interruption at some point											
	Campo Grande				Curitiba				Florianópolis			
	No	Yes	Total	p-value	No	Yes	Total	p-value	No	Yes	Total	p-value
	136 (4.1)	3,162 (95.9)	3,298 (100.0)		193 (3.6)	5,124 (96.4)	5,317 (100.0)		370 (5.1)	6,894 (94.9)	7,264 (100.0)	
Sex												
Women	35 (25.7)	958 (30.3)	993 (30.1)	0.256	27 (14.0)	1,043 (20.4)	1,070 (20.1)	0.030	93 (25.1)	2,145 (31.1)	2,238 (30.8)	0.015
Men	101 (74.3)	2,204 (69.7)	2,305 (69.9)		166 (86.0)	4,080 (79.6)	4,246 (79.9)		277 (74.9)	4,749 (68.9)	5,026 (69.2)	
Race/skin color												
White	53 (39.0)	1,422 (45.0)	1,475 (44.7)	0.181	113 (58.8)	3,153 (61.5)	3,266 (61.4)	0.670	286 (77.3)	5,336 (77.4)	5,622 (77.4)	0.954
Black	74 (54.4)	1,612 (51.0)	1,686 (51.1)		35 (18.1)	828 (16.2)	863 (16.2)		63 (17.0)	1,191 (17.3)	1,254 (17.3)	
Asian/Indigenous/not informed	9 (6.6)	128 (4.0)	137 (4.2)		45 (23.3)	1,143 (23.3)	1,188 (22.3)		21 (5.7)	367 (5.3)	388 (5.3)	
Level of education (years)												
Up to seven	25 (18.9)	939 (29.9)	964 (29.5)	0.029	18 (9.7)	530 (11.0)	548 (11.0)	0.134	46 (12.7)	1,169 (17.4)	1,215 (17.2)	0.003
Eight to 11	54 (40.9)	1,067 (34.0)	1,121 (34.3)		54 (29.2)	1,422 (29.6)	1,476 (29.6)		93 (25.6)	1,980 (29.5)	2,073 (29.3)	
12 or over	43 (32.6)	982 (31.3)	1,025 (31.3)		77 (41.6)	1,639 (34.1)	1,716 (34.4)		174 (47.9)	2,594 (38.6)	2,768 (39.1)	
Unknown	10 (7.6)	150 (4.8)	160 (4.9)		36 (19.5)	1,212 (25.2)	1,248 (25.0)		50 (13.8)	973 (14.5)	1,023 (14.5)	
Age group (years)												
15 to 29	25 (18.4)	512 (16.2)	537 (16.3)	0.652	45 (23.3)	781 (15.2)	826 (15.5)	0.012	55 (14.9)	834 (12.1)	889 (12.3)	0.178
30 to 39	41 (30.1)	852 (27.0)	893 (27.1)		55 (28.5)	1,418 (27.7)	1,473 (27.7)		106 (28.8)	1,827 (26.6)	1,933 (26.7)	
40 to 49	31 (22.8)	759 (24.0)	790 (24.0)		41 (21.2)	1,165 (22.7)	1,206 (22.7)		76 (20.7)	1,662 (24.2)	1,738 (24.0)	
50+	39 (28.7)	1,037 (32.8)	1,076 (32.6)		52 (26.9)	1,758 (34.3)	1,810 (34.1)		131 (35.6)	2,550 (37.1)	2,681 (37.0)	

Source: Brazilian Medication Logistics Control System (2023).

In the logistic models adjusted for treatment interruption, individuals aged 40 to 49 years (OR=1.63; 95%CI 1.03-2.56) and 50 years or older (OR=1.73; 95%CI 1.12-2.66) in Curitiba were more likely to interrupt treatment when compared to those aged 15 to 29 years. Regarding level of education, participants without a school certificate also had a higher likelihood of interruption (OR=1.66; 95%CI: 1.06-2.62). In Florianópolis, interruption was more frequent among individuals with up to seven years of formal education (OR=1.62; 95%CI: 1.15-2.29) and individuals with eight to 11 years of formal education (OR=1.42; 95%CI: 1.09-1.84) (Table 2).

**Table 2:** Logistics model considering treatment interruption. Campo Grande (MS), Curitiba (PR), and Florianópolis (SC), from October 2019 to September 2022.

Variables	Treatment interruption at some point											
	p-value	OR	95%CI OR		p-value	OR	95%CI OR		p-value	OR	95%CI	
			Inf.	Sup.			Inf.	Sup.			Inf.	Sup.
	Campo Grande				Curitiba				Florianópolis			
Men	0.351	0.82	0.54	1.24	0.159	0.73	0.47	1.13	0.099	0.81	0.63	1.04
Age group (years)												
15 to 29		1.00				1.00				1.00		
30 to 39	0.813	1.06	0.63	1.79	0.046	1.53	1.01	2.32	0.278	1.21	0.86	1.69
40 to 49	0.644	1.13	0.66	1.97	0.035	1.63	1.03	2.56	0.058	1.42	0.99	2.04
50+	0.756	1.08	0.64	1.85	0.013	1.73	1.12	2.66	0.233	1.23	0.88	1.71
Race												
White		1.00				1.00				1.00		
Black	0.193	0.78	0.54	1.13	0.645	0.91	0.61	1.36	0.990	0.99	0.74	1.32
Asian/Indigenous/ not informed	0.519	0.75	0.31	1.81	0.127	0.73	0.48	1.09	0.990	1.00	0.61	1.66
Level of education (years)												
Up to seven	0.089	1.59	0.93	2.73	0.401	1.26	0.74	2.15	0.006	1.62	1.15	2.29
Eight to 11	0.501	0.86	0.57	1.32	0.254	1.23	0.86	1.763	0.009	1.42	1.09	1.84
12 or over		1.00				1.000				1.00		
Unknown	0.346	0.69	0.32	1.49	0.027	1.67	1.06	2.62	0.132	1.29	0.92	1.81

OR: odds ratio; 95%CI: 95% confidence interval; Inf: inferior; Sup: superior.

Source: Brazilian Medication Logistics Control System (2023).

Regarding the frequency of interruption, we verified that in Campo Grande and Curitiba women had a higher frequency of interruption in the categories one to five and six to ten times (Campo Grande: p<0.000; Curitiba: p<0.000). In Florianópolis, the highest frequency of interruption among women occurred in the category one to five times (p=0.009). Illiterate individuals or those with unknown level of education had a higher frequency of interruption in all categories in the three municipalities (Campo Grande: p=0.002; Curitiba: p=0.003; Florianópolis: p=0.006). The age group of 15 to 29 years had a higher frequency of interruption in Campo Grande and Curitiba for the categories one to five and six to ten times (Campo Grande: p=0.002; Curitiba: p<0.000) (Table 3).

**Table 3:** Distribution of sociodemographic characteristics according to the frequency of interruption in each municipality. Campo Grande (MS), Curitiba (PR), and Florianópolis (SC), from October 2019 to September 2022.

Variables	Frequency of treatment interruption														
	Campo Grande					Curitiba					Florianópolis				
	None	One to five	Six to ten	Total	p-value	None	One to five	Six to ten	Total	p-value	None	One to five	Six to ten	Total	p-value
	136 (4.1)	2,814 (85.3)	348 (10.6)	3,298 (100.0)		193 (3.6)	4,695 (88.3)	429 (8.1)	5,317 (100.0)		370 (5.1)	6,765 (93.1)	129 (1.8)	7,264 (100.0)	
Sex															
Women	35 (25.7)	814 (28.9)	144 (41.4)	993 (30.1)	0.000	27 (14.0)	924 (19.7)	119 (27.7)	1,070 (20.1)	0.000	93 (25.1)	2,095 (31.0)	50 (38.8)	2,238 (30.8)	0.009
Men	101 (74.3)	2,000 (71.1)	204 (58.6)	2,305 (69.9)		166 (86.0)	3,770 (80.3)	310 (72.3)	4,246 (79.9)		277 (74.9)	4,670 (69.0)	79 (61.2)	5,026 (69.2)	
Race/skin color															
White	53 (39.0)	1,275 (45.3)	147 (42.2)	1,475 (44.7)	0.162	113 (58.5)	2,873 (61.2)	280 (65.3)	3,266 (61.4)	0.159	286 (77.3)	5,235 (77.4)	101 (78.3)	5,622 (77.4)	0.866
Black	74 (54.4)	1,421 (50.5)	191 (54.9)	1,686 (51.1)		35 (18.1)	776 (16.5)	52 (12.1)	863 (16.2)		63 (17.0)	1,172 (17.3)	19 (14.7)	1,254 (17.3)	
Asian/ Indigenous/not informed	9 (6.6)	118 (4.2)	10 (2.9)	137 (4.2)		45 (23.3)	1,046 (22.3)	97 (22.6)	1,188 (22.3)		21 (5.7)	358 (5.3)	9 (7.0)	388 (5.3)	
Level of education (years)															
Up to seven	25 (18.9)	812 (29.1)	127 (36.5)	964 (29.5)	0.002	18 (9.7)	478 (10.9)	52 (12.8)	548 (11.0)	0.003	46 (12.7)	1,140 (17.3)	29 (23.6)	1,215 (17.2)	0.006
Eight to 11	54 (40.9)	947 (33.9)	120 (34.5)	1,121 (34.3)		54 (29.2)	1,310 (29.8)	112 (27.7)	1,476 (29.6)		93 (25.6)	1,950 (29.6)	30 (24.4)	2,073 (29.3)	
12 or over	43 (32.6)	896 (32.1)	86 (24.7)	1,025 (31.3)		77 (41.6)	1,526 (34.7)	113 (27.9)	1,716 (34.4)		174 (47.9)	2,549 (38.7)	45 (36.6)	2,768 (39.1)	
Unknown	10 (7.6)	135 (4.8)	15 (4.3)	160 (4.9)		36 (19.5)	1,084 (24.6)	128 (31.6)	1,248 (25.0)		50 (13.8)	954 (14.5)	19 (15.4)	1,023 (14.5)	
Age group (years)															
15 to 29	25 (18.4)	483 (17.2)	29 (8.4)	537 (16.3)	0.002	45 (23.3)	757 (16.1)	24 (5.6)	826 (15.5)	0.000	55 (14.9)	824 (12.2)	10 (7.9)	889 (12.3)	0.206
30 to 39	41 (30.1)	760 (27.0)	92 (26.5)	893 (27.1)		55 (28.5)	1,334 (28.4)	84 (19.6)	1,473 (27.7)		106 (28.8)	1,796 (26.6)	31 (24.4)	1,933 (26.7)	
40 to 49	31 (22.8)	663 (23.6)	96 (27.7)	790 (24.0)		41 (21.2)	1,054 (22.5)	111 (25.9)	1,206 (22.7)		76 (20.7)	1,625 (24.1)	37 (29.1)	1,738 (24.0)	
50+	39 (28.7)	907 (32.2)	130 (37.5)	1,076 (32.6)		52 (26.9)	1,549 (33.0)	209 (48.8)	1,810 (34.1)		131 (35.6)	2,501 (37.1)	49 (38.6)	2,681 (37.0)	

**Figure 1:**
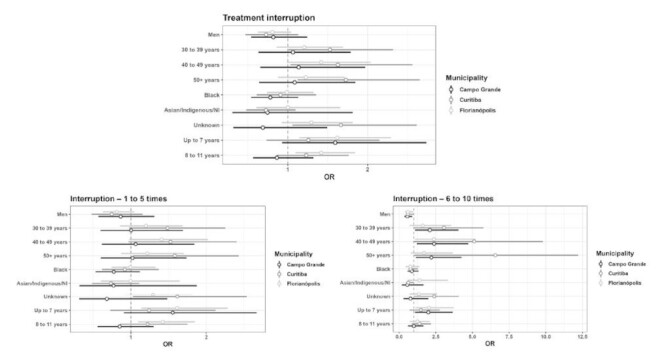
Logistics models of treatment interruption and multinomial model of interruption frequency. Campo Grande (MS), Curitiba (PR), and Florianópolis (SC), from October 2019 to September 2022.

In Figure 1 we show the results of the logistic and multinomial models for interruption and frequency of interruption in the three municipalities analyzed. Individuals aged 50 years or older in Curitiba showed a higher probability of treatment interruption (OR=1.728; 95%CI 1.124-2.655). Regarding level of education, participants with unknown education had a higher risk (OR=1.666; 95%CI 1.061-2.627), whereas in Florianópolis, participants with up to seven years (OR=1.621; 95%CI 1.146-2.293) and with eight to 11 years (OR=1.420; 95%CI 1.094-1.844) of formal education had a higher probability of interruption.

*Treatment interruption refers to interruption at any point during follow-up; the results of the interruption frequency models from one to five times and from six to ten times refer to the multinomial model and the others, to logistic models; NI: not informed; OR: odds ratio.

In the frequency of interruption of one to five times, individuals aged 50 years or older in Curitiba had a higher risk (OR=1.582; 95%CI 1.030-2.433) as well as participants with unknown level of education (OR=1.615; 95%CI 1.030-2.538). In Florianópolis, the risk was higher for participants with up to seven years of formal education (OR=1.613; 95%CI: 1.140-2.282) and for those with eight to 11 years (OR=1.423; 95%CI: 1.096-1.848) (Figure 1).

As for the frequency of interruption of six to ten times, men had a lower probability of interruption compared to women in the three municipalities (Campo Grande: OR=0.558; 95%CI 0.350-0.888; Curitiba: OR=0.598; 95%CI 0.482-0.973; Florianópolis: OR=0.613; 95%CI 0.383-0.949). In Curitiba, the risk was higher for individuals aged 50 years or older (OR=6.565; 95%CI 1.030-12.218). Regarding level of education, the risk was high for participants with up to seven years of formal education in Campo Grande (OR=1.986; 95%CI 1.081-3.650), for those with unknown level of education in Curitiba (OR=2.397; 95%CI 1.028-4.060), and for participants with up to seven years of formal education in Florianópolis (OR=2.037; 95%CI 1.114-3.723) (Figure 1).

## DISCUSSION

We found that ART interruption was higher among women and individuals with low levels of education. In Curitiba and Florianópolis, women, especially in the age groups 15 to 29 and 30 to 39 years, were more likely to abandon the treatment. Low level of education, especially up to seven years of formal education or unknown, was associated with a higher frequency of interruption in the three municipalities. Participants aged 50 years or older also had a higher risk of interruption, especially in Curitiba.

According to the results, there is a higher probability of treatment interruption among women in Curitiba and Florianópolis. Family responsibilities, gender stigma, housework overburden, and difficulties in accessing healthcare services negatively influence the continuity of treatment^7^ ^,^ ^24^ ^-^ ^26^. Authors of a systematic review highlighted that stigma related to HIV and gender barriers disproportionately affect the continuity of treatment in low- and middle-income countries, where gender inequalities are most pronounced.

The interruption of treatment for HIV/AIDS can affect men and women differently. For Boynton et al.^27^, strategies sensitive to gender and sexuality are necessary in the care of PLHIV. Conversely, Demartoto et al.^28^ emphasized that the HIV/AIDS pandemic highlighted the importance of implementing structural and cultural changes in health policies and institutional practices, aiming to strengthen the training of professionals and respond effectively to the complex and dynamic needs of PLHIV.

In this study, we showed a significant association between low levels of education and a higher probability of interruption of antiretroviral therapy, especially in Campo Grande and Florianópolis. These findings corroborate the literature, according to which level of education is an important determinant of adherence to HIV/AIDS treatment. Authors of a study conducted in Brazil showed that users with low levels of education had a significantly higher prevalence of nonadherence to ART compared to those with eight or more years of formal education^29^. Other researchers have identified that the lower the level of education, the greater the risk of interrupting treatment^30^ ^,^ ^31^. Individuals with low level of education face difficulties in understanding the therapy regimen and have less access to health information and difficulty raising awareness about the importance of ART and its benefits, such as viral suppression and prevention of transmission, thus impairing the continuity of treatment^19^ ^,^ ^29^ ^,^ ^32^.

We found no statistical significance in the analysis of treatment interruption according to race/skin color in any of the cities. This finding contrasts with other studies whose authors indicate that the Black population is at greater risk of interrupting treatment because of factors such as social inequalities, limited access to healthcare services, and stigma, as evidenced in the study by Bogart et al.^33^, developed in the United States of America between 2010 and 2012. Authors of another study carried out in the USA between 2015 and 2019 identified that, even with the adjustment of social and structural determinants of health, such as poverty, transportation needs, and level of education, racial disparities in adherence to ART still remain^34^_._

Concerning the analysis by age group, we identified that, in Curitiba, PLHIV aged between 30 and 39 years had a higher rate of treatment interruption compared to other groups. A similar result was identified by Silva et al.^29^ in a study carried out in Salvador, capital of Bahia, in which youngest individuals were 2.2 times more likely to not adhere to treatment when compared to the oldest ones. Mtisi et al.^35^ carried out a study focused on risk factors for treatment interruption among HIV-infected adolescents in Tanzania, emphasizing the impact of age on treatment continuity. Furthermore, Bansi-Matharu et al.^31^, in a multicenter study conducted in 2015, observed that users in older age groups had a reduced risk of interruptions.

Our study has limitations due to the use of secondary data. Despite the important information provided by Siclom, its data may be subject to registration errors and inconsistencies in the insertion, affecting the accuracy of the analyses. The system does not gather all relevant variables, especially those related to social factors that influence treatment interruption.

The absence of qualitative data also prevents a deeper understanding of the reasons underlying the observed patterns, suggesting the need for studies that incorporate qualitative methods and primary data for a more comprehensive analysis.

In addition, it should be considered that the retrieval of antiretroviral medication does not guarantee its effective use. The analysis based on medication dispensing records does not ensure that patients correctly followed the prescribed regimen, and there may be cases of nonadherence even after retrieving the medicine.

It is worth noting that, during the period of this analysis, the new coronavirus (COVID-19) pandemic took place, which may have impacted the results, as the pandemic affected the provision of services related to HIV/AIDS^36^.

In this study, we analyzed the sociodemographic characteristics associated with the interruption of HIV/AIDS treatment in the units of analysis, and the results point to the need for person-centered strategies adapted to each territorial context to address the underlying causes of interruption.

In Curitiba and Florianópolis, women were more prone to interruption, possibly due to barriers related to access to healthcare services and gender issues. Low level of education was also a relevant determinant in Campo Grande and Florianópolis, where greater treatment interruption was verified for individuals with lower levels of education.

The findings highlight the importance of considering territorial contexts in public policies, as generic strategies may not be effective. Factors, such as sex, race/skin color, and level of education, seem to operate together, aggravating the negative impacts on treatment continuity. Ther fore, a systemic approach, integrated and adapted to local barriers, is necessary to promote equity in access.

## RESPONSIBILITY WAIVER

This publication was produced through the Cooperation Agreement No. NU2GGH002174, signed between the Foundation for Scientific and Technological Development in Health, the Sergio Arouca National School of Public Health, from Fundação Oswaldo Cruz, and the Centers for Disease Control and Prevention of the United States of America, being funded by the United States President’s Emergency Plan for AIDS Relief. Its content is the sole responsibility of the authors and does not necessarily represent the official view of the funding party.
