# Supplementary material for: Factors associated with the interruption of antiretroviral therapy among people living with HIV/AIDS in Brazilian municipalities between 2019 and 2022
Source: Rev Bras Epidemiol. 2025 Apr 7;28:e250015. doi: 10.1590/1980-549720250015 (PMC11980824; doi:10.1590/1980-549720250015)
Supplement: Translation

# Translation: Fatores associados à interrupção do tratamento antirretroviral de pessoas que vivem com HIV/aids em municípios brasileiros entre 2019 e 2022

**DOI:** 10.1590/1980-549720250015.2

**Authors:** Ana Paula da Cunha, Jurema Corrêa da Mota, Marly Marques da Cruz, Raquel Miranda, Liza Rosso, Mariele Kruppa, Juliane Santos, Andreia Souza Pinto da Silva, Giordana Faccin, Caroline Schweitzer, Ronaldo Zonta, Vanda Lúcia Cota

**Affiliations:** I Fundação Oswaldo Cruz, Escola Nacional de Saúde Pública Sergio Arouca - Rio de Janeiro (RJ), Brasil.; II Fundação Oswaldo Cruz, Instituto de Comunicação e Informação Científica e Tecnológica em Saúde - Rio de Janeiro (RJ), Brasil.; III Secretaria Municipal da Saúde de Curitiba - Curitiba (PR), Brasil.; IV Secretaria Municipal de Saúde de Campo Grande - Campo Grande (MS), Brasil.; V Secretaria Municipal de Saúde de Florianópolis - Florianópolis (SC), Brasil.

**Keywords:** Síndrome da imunodeficiência adquirida, Terapia antirretroviral de alta atividade, Suspensão de tratamento, Cooperação e adesão ao tratamento, Barreiras ao acesso aos cuidados de saúde

## Abstract

**Objetivo:**

Analisar os fatores associados à interrupção do tratamento antirretroviral entre as pessoas que vivem com o vírus da imunodeficiência humana (HIV) participantes do Projeto A Hora é Agora nos municípios de Campo Grande (MS), Curitiba (PR) e Florianópolis (SC).

**Métodos:**

Estudo transversal, analítico, com dados do Sistema de Controle Logístico de Medicamentos coletados entre outubro de 2019 e setembro de 2022. Foram realizadas análises bivariadas para verificar associações entre sexo, faixa etária, raça/cor e escolaridade, desfechos de interrupção e número de vezes que interrompeu o tratamento, utilizando o teste χ^2^, com nível de significância de 5%.

**Resultados:**

Em Campo Grande, a interrupção do tratamento foi mais frequente entre aqueles com de oito a 11 anos de estudo (34,0%). Em Curitiba, as mulheres (20,4%) apresentaram maior frequência de interrupção do tratamento, e a faixa etária de 50 anos ou mais apresentou maior chance de interrupção do tratamento (*odds ratio* - OR=1,73; intervalo de confiança de 95% - IC95% 1,12-2,66). Em Florianópolis 31,1% das mulheres tiveram interrupção do tratamento, e a escolaridade de até sete anos apresentou maior chance de interrupção (OR=1,62; IC95% 1,15-2,29).

**Conclusões:**

A interrupção do tratamento antirretroviral esteve significativamente associada às variáveis sexo, escolaridade e faixa etária, com padrões distintos entre os contextos territoriais analisados. Esses achados ressaltam a necessidade de intervenções específicas voltadas para grupos vulneráveis, considerando as diferenças regionais e os desafios locais para melhorar a adesão ao tratamento.

## INTRODUÇÃO

Apesar dos esforços empreendidos em escala global para combater o vírus da imunodeficiência humana (HIV)/aids por meio de avanços nas estratégias de prevenção e na terapia antirretroviral (TARV), a infecção continua a representar um desafio substancial para a saúde pública em todo o mundo^1^.

O Brasil assegura o acesso universal e gratuito à TARV desde 1996^2^, sendo esta indicada para todas as pessoas vivendo com HIV (PVHIV), independentemente de sua situação imunológica. Quando administrada de maneira adequada, a TARV demonstra eficácia em suprimir a replicação do vírus^3^, no entanto ela não proporciona cura definitiva, e, em caso de interrupção do tratamento, o vírus regularmente recupera sua atividade em semanas^4^.

Desde a década de 1980 ocorreram cerca de 20,8 milhões de mortes por HIV/aids no mundo e 382.521 no Brasil^2^ ^,^ ^5^. Atualmente, cerca de 39,8 milhões de pessoas estão vivendo com a infecção/doença no mundo, e, destas, 29,8 milhões estão sendo tratadas^5^. No Brasil, existem aproximadamente 990 mil PVHIV, das quais 83% estão em tratamento, o que indica que, mesmo com o acesso universal à medicação, ainda existem pessoas que não a utilizam^5^.

Apenas a distribuição gratuita da medicação não garante o acesso ao tratamento, visto que as desigualdades no acesso e na adesão aos cuidados de saúde, o estigma associado à infecção, comportamentos de risco e desafios na implementação de estratégias eficazes de prevenção contribuem para a ocorrência da doença^6^ ^-^ ^8^.

No Brasil, foi estabelecida a Cascata do Cuidado Contínuo à PVHIV, abrangendo o diagnóstico oportuno, vinculação e retenção ao serviço, início da TARV e controle viral por meio da adesão ao tratamento^9^. Embora o uso da medicação pareça uma ação individual, a adesão envolve responsabilidades compartilhadas entre o usuário, a equipe de saúde e a rede de apoio. Essa colaboração é crucial para abordar as singularidades socioculturais e econômicas, visando melhorar a qualidade de vida das PVHIV^10^ ^,^ ^11^.

A não adesão é a principal causa de falha na TARV, ocorrendo quando o paciente não segue consistentemente o regime terapêutico, seja por não iniciar o tratamento, seja por interrompê-lo. Isso compromete a supressão viral, reduz a eficácia e aumenta o risco de resistência medicamentosa e progressão da doença^10^ ^,^ ^12^ ^,^ ^13^.

As interrupções de tratamento das PVHIV podem ser temporárias ou completas. Esses padrões podem ser influenciados por fatores sociodemográficos, comportamentais e de acesso aos serviços de saúde, levando a aumento da carga viral, progressão da doença e comprometendo o controle do HIV^14^ ^,^ ^15^.

Essas dificuldades são particularmente evidentes em cidades como Florianópolis (SC), Curitiba (PR) e Campo Grande (MS), que apresentam desafios epidemiológicos e sociais específicos, impactando diretamente a continuidade do tratamento e adesão a ele^16^ ^-^ ^18^. Florianópolis apresenta a terceira maior taxa de detecção de HIV/aids do Brasil^2^, enfrentando um cenário que exige ações contínuas de controle e prevenção^17^. Em Curitiba, os desafios de adesão ao tratamento são expressivos entre grupos vulneráveis, por causa de fatores como estigma e barreiras sociais^2^ ^,^ ^8^. Em Campo Grande, as dificuldades também são nítidas, particularmente em áreas periféricas com acesso restrito aos serviços de saúde^2^ ^,^ ^18^.

Diante da necessidade de enfrentamento da interrupção de tratamento, o Projeto A Hora é Agora (AHA) foi implementado em cinco municípios brasileiros, mas este estudo incluiu Curitiba, Porto Alegre e Florianópolis, já que Porto Alegre (RS) e Fortaleza (CE) foram implantados posteriormente e não tinham dados suficientes para o período analisado. O Projeto AHA faz parte de um acordo de cooperação entre a Escola Nacional de Saúde Pública Sergio Arouca, da Fundação Oswaldo Cruz (Fiocruz), e o Centers for Disease, Control and Prevention (CDC), financiado por meio do President’s Emergency Plan for AIDS Relief, numa parceria com o Departamento de HIV/Aids, Tuberculose, Hepatites Virais e outras Infecções Sexualmente Transmissíveis do Ministério da Saúde, as secretarias municipais de Saúde e organizações da sociedade civil. Esses parceiros institucionais constituem o comitê gestor do projeto, que tem como objetivo ampliar o acesso à prevenção, ao diagnóstico e ao tratamento imediato para o HIV/aids com foco na população-chave mais vulnerabilizada. Para tal, foram introduzidas por meio do projeto, nos municípios selecionados, algumas inovações em serviços do Sistema Único de Saúde que permitem maior agilidade na intervenção, como: carga viral e CD4 rápidos, creatinina rápida, o *linkador* (navegador de par) que apoia o(a) usuário(a) para garantir vinculação/adesão, a entrega do autoteste e do remédio em casa (TARV e PReP *delivery*), teleconsulta e telePReP. Esse projeto implementa-se no âmbito do SUS, ou seja, tem a garantia de acesso universal, contudo atua com dois componentes estratégicos, que é o da comunicação, focada na população mais vulnerabilizada que enfrenta frequentes barreiras de acesso, e o do monitoramento/avaliação, que permite acompanhamento de todas as estratégias existentes e os ajustes necessários ao longo da implementação.

A avaliação da interrupção do tratamento antirretroviral é essencial para monitorar a efetividade das políticas de saúde e identificar barreiras institucionais, como problemas de acesso e estigma, permitindo identificar padrões e subgrupos vulneráveis para que seja possível melhorar a adesão ao tratamento^19^.

Pressupôs-se que a interrupção do tratamento antirretroviral entre as PVHIV fosse influenciada por características sociodemográficas. Diante do exposto, este estudo teve como objetivo analisar os fatores associados à interrupção do tratamento antirretroviral entre as PVHIV participantes do Projeto AHA nos municípios de Campo Grande, Curitiba e Florianópolis.

## MÉTODOS

### Desenho e população do estudo

Estudo transversal e analítico, com unidades de análise nos municípios de Campo Grande, Curitiba e Florianópolis. Foram incluídas PVHIV com 15 anos ou mais, independentemente do sexo ao nascimento, que faziam uso da TARV entre outubro de 2019 e setembro de 2022, período do Projeto AHA. Os dados foram obtidos do Sistema de Controle Logístico de Medicamentos (Siclom), que permite monitorar a dispensação de antirretrovirais.

Após aprovação ética, os dados identificados foram anonimizados e armazenados de forma segura e restrita. A base de dados incluiu informações sociodemográficas e dispensações de medicamentos, com completude superior a 90%. Foram criadas variáveis para análise da interrupção do tratamento, definida pela ausência de retirada do medicamento por mais de 28 dias após a data prevista. As interrupções foram categorizadas como nenhuma, uma a cinco vezes e seis a 10 vezes.

### Desfechos

Para análise do perfil de interrupções, foram considerados como abandono todos aqueles que em algum momento do período não retiraram a medicação 28 dias após a data prevista da retirada da TARV, sendo aplicado esse critério para todos os intervalos entre as dispensações no período. Com base nisso, foram considerados dois desfechos:

- Houve interrupção de tratamento (sem dispensação de TARV) em algum momento (sim, não);
- Frequência de interrupção do tratamento no período do estudo (nenhuma; uma a cinco vezes; e seis a 10 vezes).

### Covariáveis

As covariáveis consideradas foram: sexo ao nascimento (feminino, masculino), faixa etária (15-29, 30-39, 40-49, e 50 anos ou mais), escolaridade (desconhecida, até sete anos, de oito a 11 anos e 12 anos ou mais) e raça/cor de pele (branca; negra - soma de preta e parda; amarela/indígena - soma de amarela e indígena). A agregação de preta e parda deve-se a características fenotípicas e sociais similares, enquanto a de amarela, indígena e não informada, ao baixo número de observações.

### Análise estatística

A análise descritiva e as associações foram realizadas separadamente para Curitiba, Campo Grande e Florianópolis, usando o teste χ^2^ para comparar as frequências das variáveis sociodemográficas em relação aos desfechos (significância de 5%). Essa abordagem setorial permitiu uma análise específica de cada local, evitando a avaliação conjunta de todos os participantes.

As frequências absolutas e relativas das covariáveis foram comparadas segundo os desfechos usando o teste χ^2^. Para analisar as associações, foram ajustados dois modelos de regressão logística: um binomial e um multinomial. A escolha dos modelos considerou a natureza dos desfechos e a complexidade das associações, mediante as categorias de menor risco como referência.

A regressão logística binomial foi utilizada para desfecho dicotômico, estimando a razão de chances em relação ao evento de referência. A regressão logística multinomial foi aplicada para desfechos com mais de duas categorias exclusivas, permitindo estimar as razões de chance para cada categoria. Essa abordagem proporciona uma análise mais abrangente e robusta para desfechos complexos.

A seleção inicial das variáveis para os modelos de regressão foi baseada em análise bivariada, com ponto de corte de p<0,20, para incluir covariáveis potencialmente associadas aos desfechos, mesmo sem significância a 5%. As inclusão e exclusão subsequentes das variáveis nos modelos finais consideraram o ajuste e a parcimônia, priorizando aquelas que contribuíram significativamente para explicar a variação dos desfechos, conforme o teste de Hosmer-Lemeshow.

Os resultados das regressões logísticas foram apresentados como *odds ratios* (OR) ajustados, com seus respectivos intervalos de confiança de 95 (IC95%). Todas as análises foram conduzidas na linguagem de programação R (versão 4.3.2), utilizando os pacotes ggplot2, dplyr, sjlabelled, nnet e gtsummary (https://www.r-project.org/)^20^ ^-^ ^23^.

### Aspectos éticos

O projeto de pesquisa foi submetido ao Comitê Científico dos Centros de Controle de Prevenção de Doenças, ao Comitê de Ética em Pesquisa da Escola Nacional de Saúde Pública Sergio Arouca/Fiocruz, à Comissão Nacional de Ética em Pesquisa e aos Comitês de Ética das secretarias municipais da Saúde de Curitiba, Florianópolis e Campo Grande em 2022, sendo aprovado em todas as instâncias, com Certificado de Apresentação de Apreciação Ética de número 59072922.0.0000.5240, pelo protocolo número 5.734.909/2022.

## RESULTADOS

Entre outubro de 2019 e setembro de 2022, 15.879 indivíduos estavam em tratamento para HIV/aids em Curitiba, Campo Grande e Florianópolis. Em Curitiba, 72% dos participantes eram homens e 65% se identificavam como brancos, com 52% apresentando baixa escolaridade (um a sete anos de estudo). As faixas etárias predominantes foram de 30 a 39 anos (30%) e de 40 a 49 anos (28%). No grupo que não abandonou o tratamento, a maioria era homem (76%) e branca (68%). Já entre os que o abandonaram, a proporção de mulheres foi maior (35%), assim como a de negros (38%), predominando as faixas de 15 a 29 anos e de 30 a 39 anos.

Em Campo Grande, 69% dos participantes eram homens e 60% brancos, com 55% tendo até sete anos de estudo. As faixas etárias mais comuns foram 30 a 39 anos (27%) e 40 a 49 anos (26%). No grupo que manteve o tratamento, 74% era homem e 63% branco. Entre os que o abandonaram, houve mais mulheres (38%) e negros (42%), com prevalência na faixa de 15 a 29 anos.

Em Florianópolis, 68% dos participantes eram homens e 62% brancos, e metade tinha entre um e sete anos de estudo. As faixas etárias mais frequentes foram 30 a 39 anos (29%) e 40 a 49 anos (25%). No grupo sem abandono, 73% era homem e 66% branco. Entre os que o abandonaram, a proporção de mulheres foi maior (42%) e de negros também (40%), com prevalência nas faixas de 15 a 29 anos e 30 a 39 anos.

Na análise da associação entre interrupção do tratamento e sexo, verificou-se que as mulheres em Curitiba (p=0,030) e Florianópolis (p=0,015) apresentaram maior propensão à interrupção. A baixa escolaridade também esteve associada à maior probabilidade de interrupção em Campo Grande (p=0,029) e Florianópolis (p=0,003). Em Curitiba, observou-se maior proporção de interrupção nas faixas etárias de 30 a 39 anos (27,7%) e 50 anos ou mais (34,1%) (p=0,012) (Tabela 1).

**Tabela 1:** Distribuição das características sociodemográficas segundo interrupção do tratamento em cada município. Campo Grande (MS), Curitiba (PR) e Florianópolis (SC), de outubro de 2019 a setembro 2022.

Variáveis	Interrupção de tratamento em algum momento											
	Campo Grande				Curitiba				Florianópolis			
	Não	Sim	Total	Valor p	Não	Sim	Total	Valor p	Não	Sim	Total	Valor p
	136 (4,1)	3.162 (95,9)	3.298 (100,0)		193 (3,6)	5.124 (96,4)	5.317 (100,0)		370 (5,1)	6.894 (94,9)	7.264 (100,0)	
Sexo												
Feminino	35 (25,7)	958 (30,3)	993 (30,1)	0,256	27 (14,0)	1.043 (20,4)	1.070 (20,1)	0,030	93 (25,1)	2.145 (31,1)	2.238 (30,8)	0,015
Masculino	101 (74,3)	2.204 (69,7)	2.305 (69,9)		166 (86,0)	4.080 (79,6)	4.246 (79,9)		277 (74,9)	4.749 (68,9)	5.026 (69,2)	
Raça/cor												
Branca	53 (39,0)	1.422 (45,0)	1.475 (44,7)	0,181	113 (58,8)	3.153 (61,5)	3.266 (61,4)	0,670	286 (77,3)	5.336 (77,4)	5.622 (77,4)	0,954
Negra	74 (54,4)	1.612 (51,0)	1.686 (51,1)		35 (18,1)	828 (16,2)	863 (16,2)		63 (17,0)	1.191 (17,3)	1.254 (17,3)	
Amarela/indígena/não informado	9 (6,6)	128 (4,0)	137 (4,2)		45 (23,3)	1.143 (23,3)	1.188 (22,3)		21 (5,7)	367 (5,3)	388 (5,3)	
Escolaridade (anos)												
Até sete	25 (18,9)	939 (29,9)	964 (29,5)	0,029	18 (9,7)	530 (11,0)	548 (11,0)	0,134	46 (12,7)	1.169 (17,4)	1.215 (17,2)	0,003
Oito a 11	54 (40,9)	1.067 (34,0)	1.121 (34,3)		54 (29,2)	1.422 (29,6)	1476 (29,6)		93 (25,6)	1.980 (29,5)	2.073 (29,3)	
12 ou mais	43 (32,6)	982 (31,3)	1.025 (31,3)		77 (41,6)	1.639 (34,1)	1716 (34,4)		174 (47,9)	2.594 (38,6)	2.768 (39,1)	
Desconhecida	10 (7,6)	150 (4,8)	160 (4,9)		36 (19,5)	1.212 (25,2)	1.248 (25,0)		50 (13,8)	973 (14,5)	1.023 (14,5)	
Faixa etária (anos)												
15 a 29	25 (18,4)	512 (16,2)	537 (16,3)	0,652	45 (23,3)	781 (15,2)	826 (15,5)	0,012	55 (14,9)	834 (12,1)	889 (12,3)	0,178
30 a 39	41 (30,1)	852 (27,0)	893 (27,1)		55 (28,5)	1.418 (27,7)	1.473 (27,7)		106 (28,8)	1.827 (26,6)	1.933 (26,7)	
40 a 49	31 (22,8)	759 (24,0)	790 (24,0)		41 (21,2)	1.165 (22,7)	1.206 (22,7)		76 (20,7)	1.662 (24,2)	1.738 (24,0)	
50+	39 (28,7)	1.037(32,8)	1.076 (32,6)		52(26,9)	1.758 (34,3)	1.810 (34,1)		131 (35,6)	2.550 (37,1)	2.681 (37,0)	

Fonte: Siclom (2023).

Nos modelos logísticos ajustados para interrupção do tratamento, os indivíduos de 40 a 49 anos (OR=1,63; IC95% 1,03-2,56) e de 50 anos ou mais (OR=1,73; IC95% 1,12-2,66) em Curitiba apresentaram maior probabilidade de interromper o tratamento quando comparados aos de 15 a 29 anos. Em relação à escolaridade, participantes sem declaração de nível de instrução também apresentaram maior chance de interrupção (OR=1,66; IC95% 1,06-2,62). Em Florianópolis, a interrupção foi mais frequente entre indivíduos com até sete anos de escolaridade (OR=1,62; IC95% 1,15-2,29) e entre aqueles com de oito a 11 anos de escolaridade (OR=1,42; IC95% 1,09-1,84) (Tabela 2).

**Tabela 2:** Modelo logístico considerando interrupção do tratamento. Campo Grande (MS), Curitiba (PR) e Florianópolis (SC), outubro de 2019 a setembro 2022.

Variáveis	Interrupção do tratamento em algum momento											
	Valor p	OR	IC95% OR		Valor p	OR	IC95% OR		Valor p	OR	IC95%	
			Inf	Sup			Inf	Sup			Inf	Sup
	Campo Grande				Curitiba				Florianópolis			
Sexo masculino	0,351	0,82	0,54	1,24	0,159	0,73	0,47	1,13	0,099	0,81	0,63	1,04
Faixa etária (anos)												
15 a 29		1,00				1,00				1,00		
30 a 39	0,813	1,06	0,63	1,79	0,046	1,53	1,01	2,32	0,278	1,21	0,86	1,69
40 a 49	0,644	1,13	0,66	1,97	0,035	1,63	1,03	2,56	0,058	1,42	0,99	2,04
50+	0,756	1,08	0,64	1,85	0,013	1,73	1,12	2,66	0,233	1,23	0,88	1,71
Raça												
Branca		1,00				1,00				1,00		
Negra	0,193	0,78	0,54	1,13	0,645	0,91	0,61	1,36	0,990	0,99	0,74	1,32
Amarela/indígena/não informado	0,519	0,75	0,31	1,81	0,127	0,73	0,48	1,09	0,990	1,00	0,61	1,66
Escolaridade (anos)												
Até sete	0,089	1,59	0,93	2,73	0,401	1,26	0,74	2,15	0,006	1,62	1,15	2,29
Oito a 11	0,501	0,86	0,57	1,32	0,254	1,23	0,86	1,763	0,009	1,42	1,09	1,84
12 ou mais		1,00				1,000				1,00		
Desconhecida	0,346	0,69	0,32	1,49	0,027	1,67	1,06	2,62	0,132	1,29	0,92	1,81

OR: odds ratio; IC95%: intervalo de confiança de 95%; Inf: inferior; Sup: superior.

Fonte: Siclom (2023).

Em relação à frequência de interrupção, verificou-se que em Campo Grande e Curitiba as mulheres apresentaram maior frequência de interrupção nas categorias uma a cinco e seis a 10 vezes (Campo Grande: p<0,000; Curitiba: p<0,000). Em Florianópolis, a maior frequência de interrupção entre as mulheres ocorreu na categoria uma a cinco vezes (p=0,009). Indivíduos sem instrução ou com escolaridade desconhecida tiveram maior frequência de interrupção em todas as categorias nos três municípios (Campo Grande: p=0,002; Curitiba: p=0,003; Florianópolis: p=0,006). A faixa etária de 15 a 29 anos apresentou maior frequência de interrupção em Campo Grande e Curitiba para as categorias uma a cinco e seis a 10 vezes (Campo Grande: p=0,002; Curitiba: p<0,000) (Tabela 3).

**Tabela 3:** Distribuição das características sociodemográficas segundo a frequência de interrupção em cada município. Campo Grande (MS), Curitiba (PR) e Florianópolis (SC), outubro de 2019 a setembro 2022.

Variáveis	Frequência de interrupção de tratamento														
	Campo Grande					Curitiba					Florianópolis				
	Nenhuma	1 a 5	6 a 10	Total	Valor p	Nenhuma	1 a 5	6 a 10	Total	Valor p	Nenhuma	1 a 5	6 a 10	Total	Valor p
	136 (4,1)	2.814 (85,3)	348 (10,6)	3.298 (100,0)		193 (3,6)	4.695 (88,3)	429 (8,1)	5.317 (100,0)		370 (5,1)	6.765 (93,1)	129 (1,8)	7.264 (100,0)	
Sexo															
Feminino	35 (25,7)	814 (28,9)	144 (41,4)	993 (30,1)	0,000	27 (14,0)	924 (19,7)	119 (27,7)	1.070 (20,1)	0,000	93 (25,1)	2.095 (31,0)	50 (38,8)	2.238 (30,8)	0,009
Masculino	101 (74,3)	2.000 (71,1)	204 (58,6)	2.305 (69,9)		166 (86,0)	3.770 (80,3)	310 (72,3)	4.246 (79,9)		277 (74,9)	4.670 (69,0)	79 (61,2)	5.026 (69,2)	
Raça/cor															
Branca	53 (39,0)	1.275 (45,3)	147 (42,2)	1.475 (44,7)	0,162	113 (58,5)	2.873 (61,2)	280 (65,3)	3.266 (61,4)	0,159	286 (77,3)	5.235 (77,4)	101 (78,3)	5.622 (77,4)	0,866
Negra	74 (54,4)	1.421 (50,5)	191 (54,9)	1.686 (51,1)		35 (18,1)	776 (16,5)	52 (12,1)	863 (16,2)		63 (17,0)	1.172 (17,3)	19 (14,7)	1.254 (17,3)	
Amarela/indígena/não informado	9 (6,6)	118 (4,2)	10 (2,9)	137 (4,2)		45 (23,3)	1.046 (22,3)	97 (22,6)	1.188 (22,3)		21 (5,7)	358 (5,3)	9 (7,0)	388 (5,3)	
Escolaridade (anos)															
Até sete	25 (18,9)	812 (29,1)	127 (36,5)	964 (29,5)	0,002	18 (9,7)	478 (10,9)	52 (12,8)	548 (11,0)	0,003	46 (12,7)	1140 (17,3)	29 (23,6)	1215 (17,2)	0,006
Oito a 11	54 (40,9)	947 (33,9)	120 (34,5)	1.121 (34,3)		54 (29,2)	1.310 (29,8)	112 (27,7)	1.476 (29,6)		93 (25,6)	1.950 (29,6)	30 (24,4)	2.073 (29,3)	
12 ou mais	43 (32,6)	896 (32,1)	86 (24,7)	1.025 (31,3)		77 (41,6)	1.526 (34,7)	113 (27,9)	1.716 (34,4)		174 (47,9)	2.549 (38,7)	45 (36,6)	2.768 (39,1)	
Desconhecida	10 (7,6)	135 (4,8)	15 (4,3)	160 (4,9)		36 (19,5)	1.084 (24,6)	128 (31,6)	1.248 (25,0)		50 (13,8)	954 (14,5)	19 (15,4)	1.023 (14,5)	
Faixa etária (anos)															
15 a 29	25 (18,4)	483 (17,2)	29 (8,4)	537 (16,3)	0,002	45 (23,3)	757 (16,1)	24 (5,6)	826 (15,5)	0,000	55 (14,9)	824 (12,2)	10 (7,9)	889 (12,3)	0,206
30 a 39	41 (30,1)	760 (27,0)	92 (26,5)	893 (27,1)		55 (28,5)	1.334 (28,4)	84 (19,6)	1.473 (27,7)		106 (28,8)	1.796 (26,6)	31 (24,4)	1.933 (26,7)	
40 a 49	31 (22,8)	663 (23,6)	96 (27,7)	790 (24,0)		41 (21,2)	1.054 (22,5)	111 (25,9)	1.206 (22,7)		76 (20,7)	1.625 (24,1)	37 (29,1)	1.738 (24,0)	
50+	39 (28,7)	907 (32,2)	130 (37,5)	1.076 (32,6)		52 (26,9)	1.549 (33,0)	209 (48,8)	1.810 (34,1)		131 (35,6)	2.501 (37,1)	49 (38,6)	2.681 (37,0)	

Fonte: Siclom (2023).

A Figura 1 apresenta os resultados dos modelos logístico e multinominal para interrupção e frequência de interrupção nos três municípios analisados. Indivíduos com 50 anos ou mais em Curitiba mostraram maior probabilidade de interrupção do tratamento (OR=1,728; IC95% 1,124-2,655). Em relação à escolaridade, participantes com instrução desconhecida tiveram maior risco (OR=1,666; IC95% 1,061-2,627), enquanto em Florianópolis participantes com até sete anos de estudo (OR=1,621; IC95% 1,146-2,293) e com de oito a 11 anos (OR=1,420; IC95% 1,094-1,844) apresentaram maior probabilidade de interrupção.

**Figura 1:** Modelos logísticos da interrupção do tratamento e modelo multinominal da frequência de interrupção. Campo Grande (MS), Curitiba (PR) e Florianópolis (SC), outubro de 2019 a setembro de 2022*.

*Interrupção do tratamento refere-se à interrupção em qualquer momento do seguimento; os resultados dos modelos de frequência de interrupção de uma a cinco vezes e de seis a 10 vezes se referem ao modelo multinomial e os demais a modelos logísticos; NI: não informado; OR: *odds ratio*.

Na frequência de interrupção de uma a cinco vezes, indivíduos de 50 anos ou mais em Curitiba tiveram maior risco (OR=1,582; IC95% 1,030-2,433), assim como participantes com escolaridade desconhecida (OR=1,615; IC95% 1,030-2,538). Em Florianópolis, o risco foi maior para participantes com até sete anos de estudo (OR=1,613; IC95% 1,140-2,282) e para aqueles com de oito a 11 anos (OR=1,423; IC95% 1,096-1,848) (Figura 1).

Para a frequência de interrupção de seis a 10 vezes, o sexo masculino teve menor probabilidade de interrupção em comparação ao feminino nos três municípios (Campo Grande: OR=0,558; IC95% 0,350-0,888; Curitiba: OR=0,598; IC95% 0,482-0,973; Florianópolis: OR=0,613; IC95% 0,383-0,949). Em Curitiba, o risco foi maior para indivíduos de 50 anos ou mais (OR=6,565; IC95% 1,030-12,218). Em relação à escolaridade, o risco foi elevado para participantes com até sete anos de estudo em Campo Grande (OR=1,986; IC95% 1,081-3,650), para aqueles com escolaridade desconhecida em Curitiba (OR=2,397; IC95% 1,028-4,060) e para participantes com até sete anos de estudo em Florianópolis (OR=2,037; IC95% 1,114-3,723) (Figura 1).

## DISCUSSÃO

O estudo mostrou que a interrupção da TARV foi maior entre mulheres e indivíduos com baixa escolaridade. Em Curitiba e Florianópolis, mulheres, especialmente nas faixas etárias de 15 a 29 e 30 a 39 anos, tiveram maior probabilidade de abandono. A baixa escolaridade, principalmente até sete anos de estudo ou desconhecida, foi associada à maior frequência de interrupção nos três municípios. Participantes com 50 anos ou mais também apresentaram maior risco de interrupção, especialmente em Curitiba.

Os resultados indicaram maior probabilidade de interrupção do tratamento entre mulheres em Curitiba e Florianópolis. As responsabilidades familiares, estigma de gênero, sobrecarga doméstica e dificuldades de acesso aos serviços de saúde influenciam negativamente a continuidade do tratamento^7^ ^,^ ^24^ ^-^ ^26^. Revisão sistemática destacou que o estigma relacionado ao HIV e às barreiras de gênero afetam desproporcionalmente a continuidade do tratamento em países de baixa e média renda, onde as desigualdades de gênero são mais acentuadas.

A interrupção do tratamento para o HIV/aids pode afetar de forma diferenciada homens e mulheres. Para Boynton et al.^27^, são necessárias estratégias sensíveis ao gênero e à sexualidade no cuidado às PVHIV. Em contrapartida, Demartoto et al.^28^ ressaltaram que a pandemia de HIV/aids evidenciou a importância de implementar mudanças estruturais e culturais nas políticas de saúde e práticas institucionais, visando fortalecer a capacitação dos profissionais e responder de maneira eficaz às necessidades complexas e dinâmicas das PVHIV.

O presente estudo evidenciou associação significativa entre baixa escolaridade e maior probabilidade de interrupção do tratamento antirretroviral, especialmente em Campo Grande e Florianópolis. Esses achados corroboram a literatura, que identifica o nível educacional como um importante determinante na adesão ao tratamento de HIV/aids. Estudo realizado no Brasil mostrou que os usuários com baixa escolaridade apresentaram prevalência significativamente maior de não adesão à TARV em comparação com aqueles com oito ou mais anos de estudo^29^. Demais estudos identificaram que, quanto menor a escolaridade, maior o risco para a interrupção do tratamento^30^ ^,^ ^31^. Indivíduos com baixa escolaridade enfrentam dificuldades em compreender o regime terapêutico e têm menos acesso a informações de saúde e dificuldade de conscientização sobre a importância da TARV e seus benefícios, como a supressão viral e prevenção da transmissão, prejudicando a continuidade do tratamento^19^ ^,^ ^29^ ^,^ ^32^.

A análise da interrupção do tratamento segundo raça/cor não mostrou significância estatística em nenhuma das cidades. Esse achado contrasta com outros estudos que indicam que a população negra apresenta maior risco de interromper o tratamento por causa de fatores como desigualdades sociais, acesso limitado aos serviços de saúde e estigma, conforme evidenciado no estudo de Bogart et al.^33^, desenvolvido nos Estados Unidos entre 2010 e 2012. Outro estudo também realizado nos Estados Unidos entre 2015 e 2019 identificou que, mesmo com o ajuste dos determinantes sociais e estruturais da saúde, como pobreza, necessidades de transporte e escolaridade, ainda permanecem as disparidades raciais na adesão à TARV^34^_._

A análise por faixa etária identificou que em Curitiba as PVHIV entre 30 e 39 anos apresentaram taxa mais alta de interrupção do tratamento em comparação com outras faixas. Resultado similar foi identificado por Silva et al.^29^ em estudo realizado em Salvador, capital da Bahia, onde os mais jovens apresentaram 2,2 vezes mais chances de não aderirem ao tratamento quando comparados aos mais velhos. Estudo realizado por Mtisi et al.^35^ apresentou uma pesquisa focada nos fatores de risco para a interrupção do tratamento entre adolescentes infectados pelo HIV na Tanzânia, enfatizando o impacto da idade na continuidade do tratamento. Também, estudo multicêntrico realizado em 2015 por Bansi-Matharu et al.^31^ observou que usuários em faixas etárias mais avançadas apresentaram risco reduzido de interrupções.

Este estudo apresenta limitações em razão do uso de dados secundários. Apesar das importantes informações proporcionadas pelo Siclom, seus dados podem estar sujeitos a erros de registro e inconsistências na inserção, afetando a precisão das análises. O sistema não captura todas as variáveis relevantes, especialmente aquelas relacionadas a fatores sociais que influenciam a interrupção do tratamento.

A ausência de dados qualitativos também impede uma compreensão mais profunda das razões subjacentes aos padrões observados, sugerindo a necessidade de estudos que incorporem métodos qualitativos e dados primários para uma análise mais abrangente.

Adicionalmente, deve-se considerar que a retirada da medicação antirretroviral não garante seu uso efetivo. A análise baseada nos registros de retirada da medicação não assegura que os pacientes seguiram corretamente o regime prescrito, podendo haver casos de não adesão mesmo após a retirada.

Sinaliza-se que, durante o período dessa análise, ocorreu a pandemia de COVID-19, o que pode ter impactado nos resultados, visto que a pandemia afetou a prestação de serviços relacionados ao HIV/aids^36^.

Este estudo analisou as características sociodemográficas associadas à interrupção do tratamento para HIV/aids nas unidades de análise, e os resultados apontam para a necessidade de estratégias centradas na pessoa e adaptadas a cada contexto territorial para abordar as causas subjacentes da interrupção.

Em Curitiba e Florianópolis, as mulheres apresentaram maior propensão à interrupção, possivelmente em razão de barreiras relacionadas ao acesso aos serviços de saúde e questões de gênero. A baixa escolaridade também foi um determinante relevante em Campo Grande e Florianópolis, onde indivíduos com menor nível educacional apresentaram maior interrupção.

Os achados ressaltam a importância de considerar contextos territoriais nas políticas públicas, pois estratégias genéricas podem não ser eficazes. Fatores como sexo, raça/cor e escolaridade parecem agir de forma conjunta, agravando os impactos negativos na continuidade do tratamento. Portanto, é necessária uma abordagem sistêmica, integrada e adaptada às barreiras locais, para promover a equidade no acesso.

## ISENÇÃO DE RESPONSABILIDADE

Esta publicação foi produzida por meio do Acordo de Cooperação número NU2GGH002174, firmado entre a Fundação para o Desenvolvimento Científico e Tecnológico em Saúde, a Escola Nacional de Saúde Pública Sergio Arouca, da Fundação Oswaldo Cruz, e os Centros de Controle e Prevenção de Doenças dos Estados Unidos, sendo financiada pelo United States President’s Emergency Plan for AIDS Relief. Seu conteúdo é de responsabilidade exclusiva dos autores e não representa, necessariamente, a visão oficial do financiador.
